# Vegetative and Adaptive Traits Predict Different Outcomes for Restoration Using Hybrids

**DOI:** 10.3389/fpls.2016.01741

**Published:** 2016-11-22

**Authors:** Philip A. Crystal, Nathanael I. Lichti, Keith E. Woeste, Douglass F. Jacobs

**Affiliations:** ^1^Hardwood Tree Improvement and Regeneration Center, Department of Forestry and Natural Resources, Purdue UniversityWest Lafayette, IN, USA; ^2^Department of Biology, Colby CollegeWaterville, ME, USA; ^3^Hardwood Tree Improvement and Regeneration Center, Northern Research Station, USDA Forest ServiceWest Lafayette, IN, USA

**Keywords:** discriminant analysis, ecological equivalency, ecophysiology, forest restoration, habitat differentiation, hybridization, introgression, *Juglandaceae*

## Abstract

Hybridization has been implicated as a driver of speciation, extinction, and invasiveness, but can also provide resistant breeding stock following epidemics. However, evaluating the appropriateness of hybrids for use in restoration programs is difficult. Past the F1 generation, the proportion of a progenitor’s genome can vary widely, as can the combinations of parental genomes. Detailed genetic analysis can reveal this information, but cannot expose phenotypic alterations due to heterosis, transgressive traits, or changes in metabolism or development. In addition, because evolution is often driven by extreme individuals, decisions based on phenotypic averages of hybrid classes may have unintended results. We demonstrate a strategy to evaluate hybrids for use in restoration by visualizing hybrid phenotypes across selected groups of traits relative to both progenitor species. Specifically, we used discriminant analysis to differentiate among butternut (*Juglans cinerea* L.), black walnut (*J. nigra* L.), and Japanese walnut (*J. ailantifolia* Carr. var. *cordiformis*) using vegetative characters and then with functional adaptive traits associated with seedling performance. When projected onto the progenitor trait space, naturally occurring hybrids (*J.* × *bixbyi* Rehd.) between butternut and Japanese walnut showed introgression toward Japanese walnut at vegetative characters but exhibited a hybrid swarm at functional traits. Both results indicate that hybrids have morphological and ecological phenotypes that distinguish them from butternut, demonstrating a lack of ecological equivalency that should not be carried into restoration breeding efforts. Despite these discrepancies, some hybrids were projected into the space occupied by butternut seedlings’ 95% confidence ellipse, signifying that some hybrids were similar at the measured traits. Determining how to consistently identify these individuals is imperative for future breeding and species restoration efforts involving hybrids. Discriminant analysis provides a useful technique to visualize past selection mechanisms and current variation in hybrid populations, especially when key ecological traits that distinguish progenitors are unknown. Furthermore, discriminant analysis affords a tool to assess ecological equivalency of hybrid populations and breeding program efforts to select for certain traits and monitor the amount of variability of those traits, relative to progenitors.

## Introduction

Naturally occurring hybridization between native and exotic species can dilute native gene pools and result in extinction, especially of rare species (Levin et al., 1996; Rhymer and Simberloff, 1996). Hybridization has also been implicated in development of invasiveness, as increased genetic diversity is correlated with adaptation to environmental variation (Ellstrand and Schierenbeck, 2006). Conversely, hybridization has been used to introduce anthropogenically desired traits for thousands of years (Rieseberg and Carney, 1998), and naturally occurring hybrids between susceptible, native taxa and resistant, exotic taxa may present a means to introduce resistance to exotic pests and pathogens in vulnerable species of concern (Michler et al., 2006).

Given that hybrids exist, their functionality influences the degree to which they threaten native populations and is the major factor in determining their effects on ecosystem integrity. Depending on the interplay between the fitness contributions of genetic material from both progenitors relative to the selection environment, hybrids may (1) introgress toward one of the progenitor species or (2) persist as an intermediate group, as is the case with most natural hybrid zones (Rhymer and Simberloff, 1996; Rieseberg and Carney, 1998, Campbell and Waser, 2001; Kimball et al., 2008). Alternatively, novel gene combinations or interactions among genetic contributions with selection (or polyploidy) may allow hybrids to (3) undergo speciation. Unless selection reduces genetic variability, hybrids may also (4) proliferate into a hybrid swarm characterized by high phenotypic variability among individuals with extremely diverse genotypes (Rhymer and Simberloff, 1996; Rieseberg and Carney, 1998, Soltis and Soltis, 2009). The actual trajectory taken by a particular hybrid population also depends on the demography and mating system of its progenitors. Under some circumstances stochastic breeding has the potential to swamp out fitness effects (Rhymer and Simberloff, 1996). In such cases, hybrid populations can present as trajectories 1, 2, or 4.

Identifying which trajectory a hybrid population is currently on would provide important information to evaluate how serious a threat hybridization poses to rare species (Levin et al., 1996; Rhymer and Simberloff, 1996) and overall ecosystem health. Assessing the hybrid trajectory is therefore critical to the study and management of hybridizing populations, yet is difficult to accomplish using genetic analysis alone. The percent admixture (Boecklen and Howard, 1997), class (Anderson and Thompson, 2002), or entire sequence of a hybrid individual does little to reveal heterosis (hybrid vigor) and other non-additive genetic effects (Chen, 2010). Genetic data also reveal little regarding how differences in physiology and metabolism would affect secondary metabolite production (Cheng et al., 2011) or transgressive phenotypes (Rieseberg and Carney, 1998).

Physical studies can reveal non-additive phenotypic effects, but are complicated by the variability associated with hybridization. Typically, such studies group hybrids by class and then regress the performance of different classes against their mean scores for one or several variables (Diskin et al., 2006; Crystal and Jacobs, 2014). Cheng et al. (2011) demonstrate in the context of herbivore resistance, that for any given trait, hybrid phenotypes can exceed, be intermediate to, equivalent to, or below one or both progenitors, and that the proportion of hybrid individuals expressing these trait levels varies among hybrid generations: F1, F2, BC1F1. As such, grouping hybrids by families (which may contain various hybrid classes depending on paternity, recombination, and independent assortment) or by generation for statistical analysis based on class-level trait averages is not particularly meaningful. Furthermore, the average performance of an entire class is relatively unimportant as natural selection acts at the individual level. Even in a class with poor overall fitness, natural selection may ensure that a few exceptional individuals proliferate, as in the classical examples of hybrid speciation in *Helianthus* and *Iris* (Arnold and Martin, 2010).

We present an application of multivariate discriminant analysis (Legendre and Legendre, 2003) to identify the current trajectory of a pool of hybrids through visualization of individual hybrids in the context of the progenitors’ trait-space. Discriminant analysis is particularly useful for assessing hybrid phenotypic selection mechanisms because it determines which variables from a candidate set most effectively describe differences among groups (i.e., progenitor species). Assuming the variables selected are those that selection is operating on, the ecological implications of those differences can be interpreted based on the environment that hybrids might be deployed into. While discriminant analysis has been previously used for morphometric analysis of hybrid taxonomy (Mayer and Mesler, 1993; Burgess et al., 2005, Barbour et al., 2007), it has not been used to investigate variation in functional traits that provide little *a priori* information on taxonomic status, but do pertain more directly to individual fitness (e.g., biomass or leaf area in plants; innate behaviors, pheromone production, or body mass in animals). The ability to simultaneously evaluate hybrids at multiple, often correlated traits is especially important, as fitness is not defined by a single character or by appearance *per se*, but rather by the interplay among traits that allow continued growth, survival, and most importantly, contributions to future generations.

**FIGURE 1:**
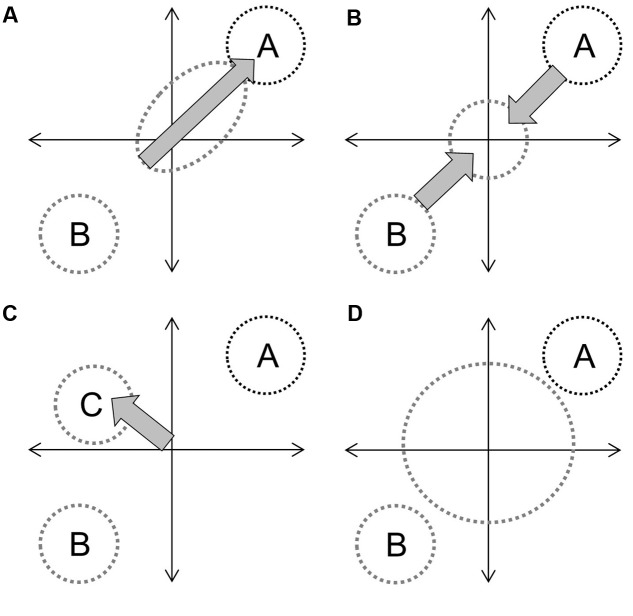
**Diagrammatic examples of four hybrid trajectories on an undefined parameter space representing combinations of functional adaptive traits: directional introgression (A)**, maintenance as an intermediate group **(B)**, speciation **(C)**, and the hybrid swarm effect **(D)**, which operates under limited or no selection. Circle size indicates overall variability and labels denote defined species. Gray arrows indicate selection, with arrow size representing effect size.

**FIGURE 2:**
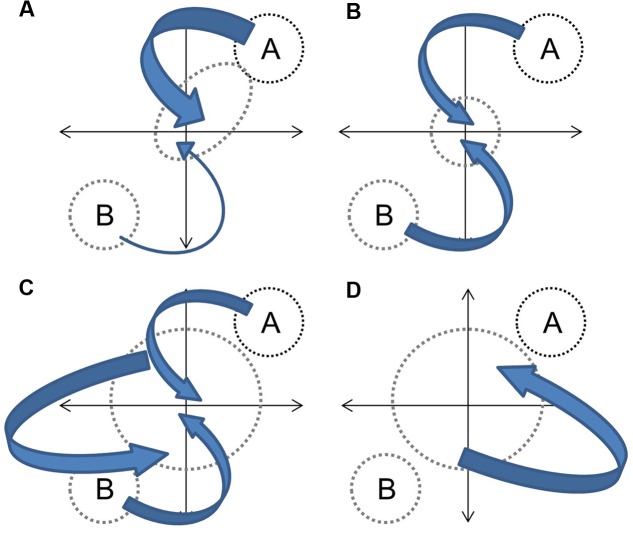
**Demography effects on hybrid phenotypes.** Independent of selection, large contributions of species A relative to species B to future generations would skew the hybrids toward species A **(A)**. Equal contributions between species A and B to future generations would pull the hybrid pool equally in both directions **(B)**. Breeding among hybrid generations and parental species would increase variability with some directionality toward progenitors **(C)**. Breeding solely among hybrids would increase variability independent of progenitor phenotypes **(D)**. Arrow size indicates size of genetic contribution.

In the context of hybridization between progenitors with distinct phenotypes, and assuming that genetic contributions are equal and that the environment is constant, the overall hybrid population would be expected to express some derivation of four basic trajectories (**Figure 1**). Directional selection toward one progenitor would result in distinct clustering of hybrids from an intermediate point between progenitors to the progenitor with greater fitness (**Figure 1A**). If both progenitors contribute equally to fitness (or if hybrids have greater fitness in an intermediate environment) the hybrid cluster will be maintained intermediate to progenitor clusters (**Figure 1B**). If hybrid speciation has occurred, the cluster will also be distinct, but may occupy a separate area of the parameter space (**Figure 1C**). Finally, if limited or no selection has occurred, or if F1 hybrid fitness facilitates the establishment of future hybrid generations (Drake, 2006), hybrids will not cluster out at all and will occupy up to the full parameter space between progenitor clusters (**Figure 1D**). They may also occupy novel trait spaces. In each instance, the size and shape of the hybrid cluster will depend upon the strength of the fitness component and the strength and direction of selection. The demography of progenitor populations, the number of hybrid generations in coexistence and their fecundity can mask selection effects (**Figure 2**).

To illustrate this approach to hybrid assessment, the trajectory of naturally occurring hybrids (*Juglans* × *bixbyi* Rehd.), between butternut (*J. cinerea* L.) and heartnut (*J. ailantifolia* Carr. var. *cordiformis*) is examined using suites of qualitative vegetative descriptors and quantitative functional adaptive traits drawn from the literature. Black walnut (*J. nigra*) was included as an outgroup to expand the trait combination space and ensure a two-dimensional ordination. Results from both trait suites are discussed in the context of the potential ecological equivalence of *J.* × *bixbyi* to butternut for conservation.

## Materials and Methods

### Study Taxa

Naturally occurring hybrids between butternut and heartnut present an ideal system to explore the use of discriminant analysis on adaptive trait variation. Heartnuts are a horticultural variety of Japanese walnut. Butternut and Japanese walnut are heterodichogamous members of the Juglandaceae, subsection Cardiocaryon (Stout, 1928; Fjellstrom and Parfitt, 1994, Kimura et al., 2003). Japanese walnut was introduced to North America in the late 1800s (Woeste and Pijut, 2009) and evidence of hybridization was first reported in 1916 (Bixby, 1919). Thus, based upon the maturation age of butternut (20 years) and Japanese walnut (10 years), it is likely that several generations of hybridization have taken place over the last century (Bonner, 2008). Hoban et al. (2009) found evidence for the existence of F1, F2, and backcross hybrids from range-wide analysis. Hybrids might be used as a source of genes for resistance to butternut canker disease caused by the suspected exotic pathogen (Furnier et al., 1999), *Ophiognomonia clavigignenti-juglandacearum* (Michler et al., 2006), which has decimated butternut populations (Ostry et al., 1994). However, Crystal and Jacobs (2014) found discrepancies between hybrids and both progenitors in terms of tolerance to flooding and drought. The extent to which the deployment of hybrids would be a practical or ecologically sensible method to address the demographic collapse of butternut is therefore unclear.

### Vegetative Characters and Functional Adaptive Traits

All four taxa were characterized at 33 vegetative morphological characters and functional adaptive traits (**Table 1**). The majority of the vegetative characters were taken from the Descriptors for Walnut (*Juglans* spp.) (IJD: IPGRI, 1994): leaf color, margin, and shape; rachis color and pubescence; average number of leaflets per leaf; and stem pubescence. To ensure distinction between *J. cinerea* and *J. ailantifolia*, three traits were chosen from Farlee et al. (2010): lenticel shape, lenticel density, and leaf scar notching. Lenticel density was determined quantitatively in a 1-cm^2^ area, between the fourth and fifth node from the shoot apex. Several other characteristics: initial shoot color and pubescence, leaf texture (glabrous, rough, or scabrous), and rachis texture (viscid or not viscid) were also assessed based on their potential to serve as vegetative descriptors or because they appeared to vary among seedlings of the taxa grown in the greenhouse.

**Table 1:** Vegetative characters and functional adaptive traits with transformations used in analysis.

Vegetative characters	Adaptive traits^∗^
Initial shoot color	^3,4^Final height
Initial shoot pubescence	^3,4^Final root collar diameter
^1^Leaf shape	Average leaf length
^1^Leaf margin	^5^Average leaf area
^1^Leaf color	^6^Average crown area
Leaf texture	Total leaf number
^1^Average number of leaflets leaf^-1∗^	^5^Estimated total leaf area
^1^Rachis color	Estimated total leaf area stem^-1^
^1^Rachis pubescence^∗^	SPAD chlorophyll content
Rachis texture	^5^Specific leaf area (SLA, m^2^ g^-1^)
^1^Stem pubescence	Photosynthesis (μmol CO_2_ g^-1^ s^-1^)
^2^Lenticel shape	SLA correlated photosynthesis (μmol CO_2_ m^-2^ s^-1^)
^2^Lenticel density^∗^	Transpiration (mmol H_2_O m^-2^ s^-1^)
^2^Leaf scar notched	Water use efficiency (umol CO_2_ mmol^-1^ H_2_O)
Number of nodes^∗^	^3,4^Root volume
	Foliar:rachis dry mass
	^3,4^Total dry mass
	^3,4^shoot:root dry mass

*Asterisks (^∗^) denote quantitative variables*. ^1^*IPGRI (1994)*, ^2^*Farlee et al. (2010)*, ^3^*Wilson and Jacobs (2006)*, ^4^*Grossnickle (2012)*, ^5^*Cornelissen et al. (2003)*, ^6^*Gauthier et al. (2013)*

The majority of functional adaptive traits were associated with seedling survival or quality assessment (Wilson and Jacobs, 2006; Grossnickle, 2012): height, root collar diameter, root volume, SPAD chlorophyll content, and dry biomass ratios of root to shoot. Height was assessed from soil medium to shoot apex. Root collar diameter was averaged from two perpendicular measurements at soil medium. Root volume was taken according to the volumetric method (Burdett, 1979) and plant material for dry biomass ratios was dried to a constant mass at 60°C. Other measures more associated with habitat type were also assessed (**Table 1**). Photosynthetic rate and transpiration were obtained on 23 and 24 August 2012 using a LI-6400 portable photosynthesis system fitted with a 6400-02B LED light source. The LI-6400 was operated under default settings with CO_2_, PAR, and temperature set to 400 ppm CO_2_, 1300 μmol m^-2^ s^-1^ PAR, and 25.0°C. CO_2_ and PAR were set high to exceed differential capabilities by species. Relative humidity in the reference chamber was maintained at 44.4–55.5%. Measurements were taken from 1100 to 1700 h as previous measurements on a subset of seedlings found no variation in photosynthetic rate from sun-up to sun-down (data not shown). Measures were logged after *A* had plateaued for at least 30 s.

### Discriminant Analysis

In discriminant analysis, each sample (individual) is assigned to a predefined group, in this case a progenitor species or an outgroup. Variables are then analyzed for differences among pre-defined groups and eigenvectors (eigenvector if *n* = 2) that maximize the among group variance are generated, with the constraints that each eigenvector must be a linear combination of the sample traits, and that all eigenvectors must be orthogonal to each other. The first step functions similarly to a multivariate analysis of variance and the second is similar to principle correspondence analysis (Legendre and Legendre, 2003). Discriminant analyses were performed using the *lda* function in the MASS package in R (Venables and Ripley, 2002; R Core Team, 2012).

To determine the hybrid trajectory, a discriminant analysis was first performed on open-pollinated individuals of the three walnut species (*J. cinerea, J. ailantifolia, and J. nigra*) using the vegetative or adaptive trait suite. Data from these individuals were then plotted on the discriminant axes and each species cluster was enclosed within a 95% confidence ellipse (*confidenceEllipse* function in the car package: Fox and Weisberg, 2011). Data from sampled hybrids were then projected onto the species biplot to visualize their location on the discriminant axes (Barbour et al., 2007).

Prior to discriminant analysis, quantitative variables were visually inspected for normality using histograms. Only shoot:root dry mass required transformation (√[shoot:root+1]). Variables were then standardized to *z*-scores to place traits on a single common scale. Individuals with missing trait values (*J. nigra* = 2, *J.* × *bixbyi* = 4) were excluded from analysis. Five *J.* × *bixbyi* individuals included in the adaptive trait projection were excluded from the vegetative projection. Due to transgressive rachis pubescence in hybrids, this trait was encoded as a quantitative variable in the vegetative character analysis. As traits in both analyses were carefully chosen *a priori*, variables were not assessed for differences among pure species using separate analysis of variance tests prior to running the discriminant function (Legendre and Legendre, 2003).

### Plant Material and Planting

Seeds from *J. cinerea*, *J. nigra*, and *J.* × *bixbyi* were collected in fall, 2011 from provenances archived at research plantations and from naturally occurring trees throughout Indiana, USA (**Table 2**). *J. ailantifolia* var. *cordiformis* seed from six of the most common cultivars (**Table 2**) was obtained from Grimo Nut Nursery (Ontario, Canada, 43° 15′ N, 79° 09′ W). After collection, seeds were hulled, washed, and allowed to air dry. On December 19, 2012 (*J*. *cinerea*, *J. nigra*, and *J.* × *bixbyi*) and December 20, 2012 (*J. ailantifolia*) seeds were rinsed in cold water, packed into bags filled with moistened, sifted peat moss by family, and cold stored at 5°C at the Purdue University Wright Forestry Center (West Lafayette, IN, USA, (40° 25′ N, 87° 02′ W). For measurement of seed characteristics (data not shown), on April 18, 2012 all seeds were unpacked, rinsed in cold water to remove excess peat, and returned to cold storage.

**Table 2:** Family, provenance, and number of sample replicates for the four taxa included in the experiment: *Juglans ailantifolia* Carr. var. *cordiformis* (family = 6: total *n* = 21), *J. cinerea* L. (8: 37), *J. nigra* L. (11: 39), and *J.* × *bixbyi* Rehd. (16: 79).

Taxon	Family	Provenance	*n*^1^
*Juglans ailantifolia* Carr. var. *cordiformis*	Bates	–	4
	Simcoe 8-2	–	6
	Fodermaier	–	5
	Campbell CW1	–	3
	Imshu	–	1
	Locket	–	2
*Juglans cinerea* L.	712	Whitewater, WI, USA	3
	717	Whitewater, WI, USA	2
	719	Whitewater, WI, USA	10
	724	Whitewater, WI, USA	5
	741	Whitewater, WI, USA	5
	766	Whitewater, WI, USA	2
	784	Plymouth, IN, USA	7
	927	Laona, WI, USA	1
	1622	Pembine, WI, USA	2
*Juglans nigra* L.	502	KS	4
	504	IA	6
	513	IA	8
	514	KS	1
	516	KY	5
	517	IA	6
	520	IA	2
	525	IA	2
	527	IL	1
	528	IL	3
	529	Unknown	1
*Juglans × bixbyi* Rehd.	701	Rochester, IN, USA	2
	735	Sanford, ME, USA	4
	745	Scotland, ON, Canada	4
	780	Plymouth, IN, USA	4
	781	Plymouth, IN, USA	7
	782	Plymouth, IN, USA	3
	803	Angola, IN, USA	5
	890	Twinsburg, OH, USA	8
	1001	Clermont, KY, USA	2
	1061	West Lafayette, IN, USA	9
	1062	Culver, IN, USA	6
	1064	West Lafayette, IN, USA	9
	1065	West Lafayette, IN, USA	7
	1066	West Lafayette, IN, USA	6
	1093	Augusta, MI, USA	3

^1^*Half-sib individuals represented in each family*.

To account for both interfamilial and species variation in seedling emergence time and the subsequent effects on growth (Seiwa, 2000), stratification was extended past the recommended 90–120 days for *J*. *cinerea* and *J. nigra* (Bonner, 2008) to 126 (*J*. *cinerea*, *J. nigra*, *J.* × *bixbyi*) and 125 (*J. ailantifolia*) days. To further minimize emergence time variation and avoid potting unviable seed, all material was pre-germinated following the methods of Woeste et al. (2011). Seeds were sown in flats containing Scotts Metro-Mix 560^®^ with Coir (MM 560) growing media and placed in the greenhouse set to maintain 26.7/18.3°C (day/night) on April 23, 2012. Seeds were removed and re-sown, every 3 days to visualize emergence. Seeds with radicle emergence less than 5 mm were wrapped in wet burlap to prevent desiccation and returned to the cooler at 5°C.

On May 22, 2012, seeds were planted as a completely randomized design into the top 7 cm of 9.63-L pots (TP818, Stuewe and Sons, Tangent, OR, USA), pre-filled with 3783.9 ± 184.1 g of MM 560 mixed with 48.2 g of Osmocote Plus^®^ 15-9-12, 8–9 month controlled-release fertilizer. Pots were immediately watered twice to allow for soil settling and prevent seed desiccation. Emergence occurred from May 29 to June 2, 2012, after which, non-germinating seedlings were discarded. Until emergence, pots were lightly watered each day to ensure the top of the pot (and the seed) remained moist; watering then occurred every 2–3 days to maintain field capacity. On July 6, 2012, chlorophyll mutants were also discarded. Seedlings were moved from benches to a cinderblock grid on the floor from July 28 to 29, 2012. Defoliation occurred from 15 to 21 September 2012, after which watering ceased. Plants were destructively harvested for biomass measurements and root volume on November 2, 2012. From May 22 to September 21, 2012, average temperature and relative humidity were (mean ± SD): 25.7 ± 6.1°C and 30.1 ± 7.1%.

## Results

**FIGURE 3:**
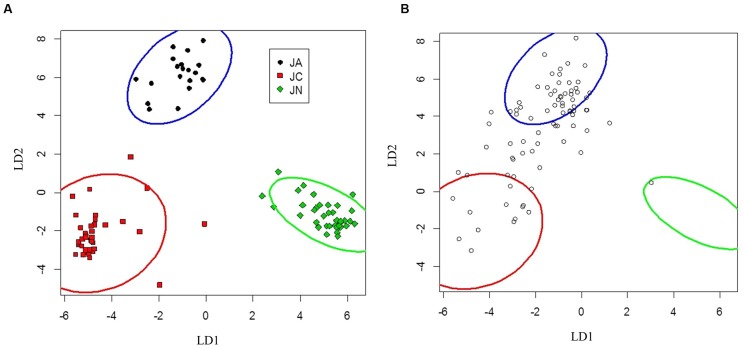
**Canonical discriminant analysis separating *Juglans ailantifolia* Carr. var. *cordiformis* (JA), *J. cinerea* L. (JC), and *J. nigra* L. (JN) based upon vegetative characteristics (A)**. *J.* × *bixbyi* Rehd. (JX) individuals (open circles) were applied to the parameter space using the discriminant function determined from **(A)**. **(B)** Pure species are enclosed by 95% confidence interval ellipses. LD1 and LD2 refer to the first and second discriminant axes, respectively.

**FIGURE 4:**
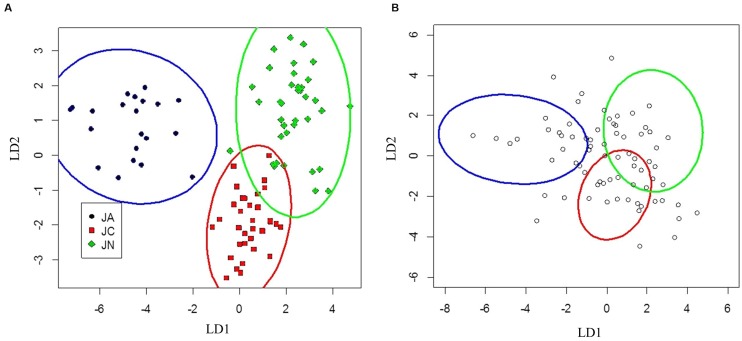
**Canonical discriminant analysis separating *J. ailantifolia* Carr. var. *cordiformis*, *J. cinerea* L., and *J. nigra* L. based upon functional adaptive traits (A)**. *J.* × *bixbyi* Rehd. individuals were applied to the parameter space as **Figure 3** **(B)**.

Seedlings of *J. ailantifolia*, *J*. *cinerea*, and *J. nigra* were effectively differentiated by the eigenvectors generated from both the vegetative and adaptive discriminant analyses (**Figures 3** and **4**). The two eigenvectors for each analysis described essentially all of the variation in their respective datasets (proportion of trace: **Table 3**). Trait loadings were relatively high for two vegetative traits: initial shoot color and leaf texture (**Table 3**). No adaptive traits were exceptional at discriminating species, with total biomass describing the most variation across both linear discriminant analyses (LDAs, **Table 3**).

**Table 3:** Loading values for vegetative and adaptive traits as assigned by canonical discriminant analysis.

Vegetative trait loadings^1^	LD1	LD2	Adaptive trait loadings^2^	LD1	LD2
Average leaflet number	0.056	-0.231	Height	0.857	-1.692
Number of nodes	0.090	0.335	Diameter	-0.994	-1.380
Lenticel density	-0.099	0.014	Average leaf length	-0.257	0.799
Rachis pubescence	0.006	0.020	Average crown area	0.455	-0.103
Initial shoot (IS) color intermediate	0.097	-1.037	Leaf number	1.073	-0.276
IS color red	-2.309	-4.228	Est. total leaf area	-0.764	-0.309
IS pubescence no	-0.651	-1.372	Leaf area stem^-1^	0.481	-0.501
IS pubescence intermediate	0.009	-1.139	Average leaf area	0.231	-0.622
IS yes	1.702	-1.783	^1^SPAD	0.130	-0.379
Leaf shape broad elliptic	0.846	-0.717	Specific Leaf Area (SLA)	1.026	-1.288
Leaf margin dentate	0.352	-0.013	SLA correlated *A*	-0.357	1.431
Leaf color green	0.796	-0.102	Photosynthetic assimilation (*A*)	0.615	-0.017
Leaf color dark green	1.226	-0.244	Transpiration (*E*)	-0.381	-1.036
Leaf texture intermediate	-2.886	1.905	Water Use Efficiency (*A/E*)	-0.241	-0.471
Leaf texture scabrous	-2.734	2.907	Root Volume	1.117	-0.589
^3^Rachis color green	0.356	-0.387	Foliar:Rachis biomass	0.295	-0.387
^3^Rachis color yellow	-0.500	0.086	Total Biomass	-1.027	2.878
^3^Rachis color red	-0.627	0.209	Sqrt(Shoot:Root + 1)	-1.450	1.248
^3^Rachis with viscid hairs	-0.002	-0.056			
^3^Lenticels striated and round	0.185	-1.519			
^3^Lenticels round	0.546	-3.534			
^3^Leafcar notched	1.979	1.121			
^3^Stem glabrous	0.094	0.740			
Proportion of trace	0.647	0.353		0.770	0.230

^1^*SPAD meter reading of chlorophyll fluorescence. Initial shoot was abbreviated IS.*

Seedlings of *J. ailantifolia*, *J*. *cinerea*, and *J. nigra* clustered tightly in the vegetative trait LDA (**Figure 3A**). There was no overlap among 95% confidence ellipses, and *J. ailantifolia*–*J*. *cinerea* were separated the least by the canonical discriminant functions, perhaps reflecting taxonomic proximity. The *J*. *cinerea* ellipse (**Figure 3A**) had the greatest dispersion overall, potentially reflecting larger genetic variation among sampled individuals relative to the other species. When hybrids were applied onto the parameter space, most individuals fell on a gradient between *J. ailantifolia* and *J*. *cinerea* ellipses (**Figure 3B**). Roughly 50% of the hybrids fell within the *J. ailantifolia* ellipse, with 21% in the *J*. *cinerea* ellipse, and 23.5% intermediate in a space not occupied by either progenitor species’ ellipse (**Table 4**).

**Table 4:** Proportion of *J.* × *bixbyi* Rehd. individuals assigned to the parameter space of *Juglans ailantifolia* Carr. var. *cordiformis*, *J. cinerea* L., and *J. nigra* L. based on the vegetative or functional adaptive trait discriminant analyses.

Parameter space	Adaptive LDA	Vegetative LDA
*Juglans ailantifolia* Carr. var. *cordiformis*	0.233	0.506
*Juglans cinerea* L.	0.287 (0.164)	0.210
Intermediate	0.069 (0.014)	0.235
*Juglans nigra* L.	0.366 (0.233)	0.012
Novel combination	0.178	0.037

*Intermediate refers to parameter space between J. ailantifolia and J. cinerea 95% confidence ellipses (CEs). Novel combination refers to parameter space not occupied by J. ailantifolia, J. cinerea, or J. nigra CEs. For the adaptive LDA, values do not sum to one due to the overlap of J. cinerea and J. nigra CEs. The overlap influenced J. cinerea, J. nigra, and Intermediate proportions. The value in parenthesis represents the proportion of hybrids assigned to that parameter space only.*

Clusters for *J. ailantifolia*, *J*. *cinerea*, and *J. nigra* based on functional adaptive traits were fairly distinct and reasonably well separated (**Figure 4A**). There was some overlap between *J*. *cinerea* and *J. nigra* ellipses, with both equivalent distances from the *J. ailantifolia* ellipse. Hybrids projected onto the parameter space were widely and relatively evenly dispersed (**Figure 4B**). The largest proportion (36.6%) of hybrids fell within the *J. nigra* ellipse including the overlap with the *J*. *cinerea* ellipse. If the combined proportion of individuals occupying the overlap between the *J*. *cinerea* and *J. nigra* ellipses were excluded, most 23.3% of the total hybrids occupied the *J. ailantifolia* parameter space (**Table 4**). Only 16.4% (28.7%, including *J*. *cinerea*/*J. nigra* overlap space) of hybrids fell within the *J*. *cinerea* ellipse. Furthermore, 17.8% of hybrids represented novel combinations of progenitor traits and fell outside the parameter space occupied by *J. ailantifolia*, *J*. *cinerea*, and *J. nigra* ellipses.

Hybrid projections in the vegetative and adaptive trait LDAs were not synonymous. Of the 73 hybrids remaining after exclusion of individuals with missing data points in the adaptive analysis, only 11 (15.1%) fell within *J. ailantifolia* space and 4 (5.5%) fell within *J*. *cinerea* space in both analyses. Half-sib progenies were fairly consistent in agreement across the two analyses, but the proportion of assignments within each family was highly variable (**Table 5**). Projections of only hybrids exhibiting initial stem color (red), the trait with the highest loading and corresponding to *J*. *cinerea* phenotype, did not all fall within *J*. *cinerea* parameter space in the functional adaptive trait analysis. Of these 13 individuals four fell within *J*. *cinerea* adaptive trait parameter space; four were outside *J. ailantifolia*, *J*. *cinerea*, and *J. nigra* parameter space; and the remaining five within *J. nigra* (2), *J. ailantifolia* (2), and intermediate *J. ailantifolia*/*J*. *cinerea* space (1).

**Table 5:** *Juglans* × *bixbyi* Rehd. family breakdown of parameter space assignments based upon the vegetative and adaptive trait discriminant analyses.

Family^1^	Vegetative		Adaptive	
129	Outside	2/4	*J*. *cinerea*	3/4
165	*J*. *cinerea*	2/4	*J*. *cinerea*	4/4
780	*J. nigra*	3/4	*J. ailantifolia*/Int.	2/4
781	*J. ailantifolia*	3/7	*J. ailantifolia*	5/7
782	Outside	2/3^2^	*J*. *cinerea*/Int./*J. ailantifolia*	1/3
803	*J. ailantifolia*	2/4^2^	*J. ailantifolia*	4/4
1001	*J. ailantifolia*/*J. nigra*	1/2	*J. ailantifolia*	2/2
1062	*J. ailantifolia*	2/6	*J. ailantifolia*/Int./*J*. *cinerea*	2/6
1093	*J. ailantifolia*/Int.	1/3^2^	*J*. *cinerea*/*J. ailantifolia*/Outside	1/3
701	Outside	2/2	Outside/*J. ailantifolia*	1/2
890	*J. ailantifolia*/*J. nigra*	3/6^3^	*J. ailantifolia*	4/8
1033	*J*. *cinerea*/*J. nigra*	2/4	*J. ailantifolia*	4/4
1061	*J*. *cinerea*/*J. nigra*	4/9	*J. ailantifolia*/Int.	4/9
1066	*J. ailantifolia*	3/6	*J. ailantifolia*	4/6
1064	*J*. *cinerea*/Outside	2/8	*J. ailantifolia*	5/8
1065	*J*. *cinerea*	4/7	Int.	4/7

^1^*See **Table 2** for family provenance*. ^2^*One individual excluded due to missing data*. ^3^*Two individuals excluded due to missing data*.

## Discussion

### Implications of Hybrid Projections on Hybrid Trajectory

Hybridization has been described as evolution in action, and it can be thought of in terms of Hardy–Weinberg principles (Anderson and Stebbins, 1954). Hybridization events introduce variability (increase cluster volume in trait space), similar to mutation, while selection acts on variability affecting cluster volume and trajectory. Unequal contributions to future generations by progenitor and hybrid populations skew genetic contributions independent of selection, similar to drift. Roughly equivalent contributions to future generations can result in a highly diverse gene pool that would have potential to evolve under selection pressure. Our example in *Juglans* demonstrates how discriminant analysis can be used to visualize the current hybrid trajectory based upon individual hybrid responses relative to the progenitor species’ responses. Without an assessment of current demography and selection pressures, however, it is not possible to parse out factors associated with hybridization, notably genetic, and demographic swamping (Todesco et al., 2016) that determine trajectory. Independent of the underlying cause(s), the current trajectory informs managers of the variability of current hybrids. Use of discriminant analysis of phenotypes, especially with non-destructive trait sampling, will allow managers to evaluate breeding and planting efforts as indicated in the Wayne and Schaffer (2016) decision tree analysis of hybrids.

Projecting *J.* × *bixbyi* hybrids onto the parameter space from the vegetative and functional adaptive discriminant analysis revealed two different trajectories. The vegetative analysis indicated that most hybrids were on Trajectory 1, introgressing toward *J. ailantifolia* (**Figure 3B**; **Table 4**). As few individuals fell onto parameter space not occupied by *J*. *cinerea*, *J. ailantifolia*, or intermediate to the two, simple removal of Japanese walnut and *J. ailantifolia*-like hybrids (Allendorf et al., 2001) could shift the pool toward the then demographically more abundant butternut. When projected on the adaptive trait discriminant analysis parameter space, however, hybrids followed no clear pattern (**Figure 4B**); they were more consistent with Trajectory 4 than Trajectory 1. Many individuals fell outside the progenitors’ or the intermediate parameter space, indicating that they represented novel phenotypes (**Table 4**). Interestingly, the hybrid cluster expanded more in the direction of black walnut (northeast: **Figure 4B**) than it did to the southeast. This possibly reflects better representation of hybrid phenotype combinations in this region of the parameter space and could indicate that additional outgroups, like Persian walnut (*J. regia* L.) would reveal even greater variation in hybrids. Trajectory 1 and 4 indicate that the use of hybrids in butternut restoration should be carefully weighed, as the phenotypes of hybrids are widely variable and probably unpredictable, even in uniformly mesic conditions. Assuming that the chosen functional traits adequately describe the autecological differences between butternut and heartnut, the data shows that only 28.7% of hybrids might match the physiology of *J*. *cinerea*. On the other hand, 23.3% of hybrids would match the physiology of *J. ailantifolia* on those sites, leading to survival in scenarios where butternut might not survive, e.g., flood (Crystal and Jacobs, 2014), and altering the trajectory of hybrids further. Furthermore, the functional variability of the hybrids increases the likelihood they will be able to colonize new areas, potentially those currently occupied by black walnut, as indicated by hybrid occupation of the *J. nigra* parameter space (**Figure 4**; **Table 4**), or become invasive (Ellstrand and Schierenbeck, 2006).

The *J.* × *bixbyi* hybrid swarm in **Figure 4** fits with current information regarding hybrid butternut demography. Woeste and Pijut (2009) report that F1 heterosis led to artificial selection of hybrids by homesteaders to serve as nut trees. Increased fitness in the F1 generation has been implicated in the establishment of future hybrid generations (Drake, 2006), as Hoban et al. (2009) found. Furthermore, the majority of these hybrids occur in anthropogenically disturbed habitats (Hoban et al., 2012a), probably due to human mediated transport of F1 individuals (Woeste and Pijut, 2009) and the short dispersal distance (generally < 100 m) of butternut seeds from mother trees (Hoban et al., 2012b) in subsequent F2 and backcross generations.

Discrepancies between the two analyses in the shape of the *J. ailantifolia*, *J*. *cinerea*, and *J. nigra* clusters are expected. The vegetative characters were chosen specifically for their power to distinguish the species. Adaptive traits were not chosen to distinguish among species and there is more variation inherent in quantitative compared to qualitative traits. This is shown by the overlap between *J*. *cinerea* and *J. nigra* clusters in the adaptive trait LDA; nevertheless, distinct clustering among species still occurred. While cluster overlap could indicate that *J*. *cinerea* and *J. nigra* are more related in terms of competition and site preference than heartnut, all seedlings in this study were grown under mesic conditions in which *Juglans* spp. typically thrive (Goodell, 1984). These taxa would likely respond differentially to drier or wetter conditions (Crystal and Jacobs, 2014), which would result in greater cluster differentiation.

The hybrid swarm effect typically establishes under little or no selection. As the majority of hybrids are found in areas near human habitation (Hoban et al., 2012a), which are usually mesic and favorable to most walnut species (Goodell, 1984), fitness effects from differences in progenitor habitat tolerance (e.g., Crystal and Jacobs, 2014) are limited. Similarly, butternut canker disease is a weak pathogen and takes years to cause mortality (Ostry et al., 1994). As such, increased fitness associated with greater disease resistance (Orchard et al., 1982) is not likely to facilitate rapid introgression of Japanese walnut alleles. The long lifespan of forest trees reduces the probability that small fitness contributions will spread rapidly throughout the population (White et al., 2007). As mature butternuts continue to succumb to age and disease, the effects of the pathogen, which is particularly lethal to seedlings (Tisserat and Kuntz, 1982), may act to select for more disease resistant hybrids that are also likely to contain more Japanese walnut genome. The hybrid swarm, however, has already established, so demographic collapse of butternut does not guarantee that hybrids will introgress toward Japanese walnut. As Allendorf et al. (2001) suggest, all progeny from hybrids are hybrids. Without sufficient numbers of progenitors, hybrids will be unlikely to ever introgress enough of the genome of butternut or Japanese walnut to be considered equivalent to one of the progenitor species in the current selection environment (Rhymer and Simberloff, 1996; Rieseberg and Carney, 1998).

Irrespective of trajectory (**Figures 3B** or **4B**), the observation that even hybrids from the same half-sibling progeny varied widely for both trait suites (**Table 5**) and presented constellations of traits unlike either progenitor indicates that general broadcasting of hybrid trees for use in lieu of butternut should be avoided (Michler et al., 2006). High variability among hybrids in functional adaptive traits indicates that extreme care should be taken in choosing individuals for a resistance breeding program. Based upon recombination and independent assortment, certain resistant trees could contribute high amounts of heartnut genome. Accordingly, for so-called butternut hybrids, a white list of seedlings screened in some fashion before outplanting, is probably most appropriate. Discriminant analysis may prove useful in this regard. Through assessment of functional adaptive traits of butternut and heartnut, the traits most responsible for differences between the taxa could be identified and utilized for hybrid screening.

### Use of Discriminant Analysis to Assess Other Unknown Hybrid Gene Pools

As Paris and Windham (1988) reported in their morphometric analysis of fern taxa, discriminant analysis is effective at identifying hybrids. Their hybrids, however, were morphologically intermediate, similar to results from F1 eucalypt hybrids (Barbour et al., 2007). As demonstrated here, discriminant analysis can be used on known hybrids to help evaluate the trajectory of the current hybrid population. Introgression (Zalapa et al., 2009) and hybrid swarm effects can be revealed with detailed genetic analysis, especially with the advance of sequencing technology to produce the required amount of markers (Boecklen and Howard, 1997; Elshire et al., 2011). Yet, genetic analysis often cannot reveal phenotype, which is ultimately what selection acts upon.

Discriminant analysis provides a relatively quick assessment of the current hybrid trajectory and, through that, historical selection mechanisms. If paired with detailed genetic analysis, it may provide answers to the difficult question commonly raised in management of hybrids: what proportion of one progenitor’s DNA indicates that a hybrid can be considered a member of a progenitor species (Allendorf et al., 2001)? It may also assist in demonstrating which portions of the genome are integral to ecological similarity, similar to studying the genomics of adaptation. In order to provide answers to these questions, however, assessment has to occur at an appropriate suite of traits. The selection of traits to include in the analysis ultimately depends on the taxa in question, desired traits, known or hypothesized selective pressures, and the environment that hybrids usually occupy. As such, it is not possible to provide all of the traits necessary for a complete assessment across all taxa, but general guidelines are presented below.

Ecological equivalence plays an important role in evaluating hybrids for conservation, as detailed by Wayne and Schaffer (2016). The assessment of hybrids for traits not directly associated with fitness but related to ecosystem function, e.g., tannins in leaves (Whitham et al., 2006) can be useful for determining the ecological equivalence of hybrids in communities. The analysis of quantitative traits as an indication of the extent of genetic introgression in the offspring of phenotypically distinct progenitors is analogous to the use of neutral genetic markers, but as Howe et al. (2003) show, populations not diverged at neutral markers often are diverged at adaptive traits. The direct assessment of functional traits is therefore critical to determine ecological similarity.

The selection of traits for analysis depends on knowledge of progenitors’ ecological function. When a particular trait is known to be essential for functionality, multivariate techniques are not essential for assessing hybrid ecological similarity. This is commonly the case for natural hybrid zones, where discrepancies in adaptive trait variation are suspected due to existing environmental gradients (Campbell and Waser, 2001; Kimball et al., 2008). Discriminant analysis at traits that have no obvious adaptive importance, however, may provide additional information on the overall variability in hybrid phenotypes relative to progenitors. For example, Lilly et al. (2012) evaluated loblolly (*Pinus taeda* L.) × shortleaf (*P. echinata* Mill.) F1 hybrids at several functional traits. They suggested that the hybrid’s reduced basal crooking, a trait associated with sprouting following fire and highly expressed in shortleaf pine, maintained the genetic integrity of shortleaf pine under a fire regime. Now that fire is essentially absent from the ecosystem, other traits govern hybrid fitness relative to progenitors. Tauer et al. (2012) report loss of genetic integrity in the demographically reduced shortleaf pine. In this example, discriminant analysis on the traits used in Lilly et al. (2012) could have revealed phenotypic covariance of traits or better explained how F1 hybrids related to progenitors.

Discriminant analysis is most useful when there is little information on progenitor ecological function. It can demonstrate how hybrids relate to progenitors across a wide suite of traits and potentially, what traits distinguish the two progenitors under a particular selection regime. This is especially important for analysis of hybrids between native and exotic congeners, which may have considerable overlap in their ecological function. As demonstrated here, combinations of many factors, not a single trait, defined differences between butternut and Japanese walnut.

Ultimately, fitness is governed by survival and contributions to future generations. Trees in this study were grown under low stress conditions and only chloroplast mutants, which would be less fit in any environment, were removed. As such, no selection occurred, an issue with most hybrid studies (Campbell and Waser, 2001). The growing environment, however, reflected current hybrid conditions. Instituting an environmental selection factor could result in greater separation of the progenitors in a discriminant analysis because of differential response to stress. It may also allow greater visualization of hybrid phenotypes as some transgressive traits are only expressed under certain conditions (Fritz et al., 2006; Ma et al., 2010). If the use of hybrids is essential to rare species conservation (e.g., for the introduction of resistance genes, Michler et al., 2006), identification of differential environmental selection mechanisms is critical to removing hybrids that do not replicate the desired progenitors’ ecological function. In the above example with loblolly and shortleaf pine hybrids, discriminant analysis at the predefined traits could be used to assess trajectory (and thus similarity) of naturally occurring progeny under the particular selection regimes of the habitat they are found in (i.e., a habitat typically associated with shortleaf or loblolly pine).

This approach to trajectory assessment is most useful for analysis of naturally occurring hybrids or later generation artificial hybrids. It bypasses the need to separate individuals into classes, which are highly variable (Cheng et al., 2011), through genotyping, which is expensive and time consuming. It can only be applied to species that are amenable to growth in a common garden, as environmental variation will lead to phenotypic variation. Multiple common garden tests can be used to plot how the hybrid trajectory will change depending on the selection environment. Gains realized from discriminant analysis are most appreciated in long-lived species (i.e., perennial plants, forest trees, and certain fish species) where direct assessment of fitness through fecundity is exceptionally tedious, and determining ecological similarity for breeding efforts extremely useful (Woeste et al., 1998). For short-lived species (i.e., annual plants, some insects, or other species with rapid generation turnover) where direct assessment of fitness can be measured through reproductive potential, discriminant analysis is not necessary. Clearly, the applicability of this technique needs to be assessed under multiple selection gradients and paired with genetic analysis to determine overall hybrid function.

## Author Contributions

PC designed and installed the experiment, collected and analyzed the data, and co-wrote the paper. NL contributed to data analysis and interpretation, and co-wrote the paper. KW assisted with data interpretation and co-wrote the paper. DJ supervised the research, helped to design the experiment, and co-wrote the paper.

## Conflict of Interest Statement

The authors declare that the research was conducted in the absence of any commercial or financial relationships that could be construed as a potential conflict of interest.
